# Developing preclinical models of neuroblastoma: driving therapeutic testing

**DOI:** 10.1186/s42490-019-0034-8

**Published:** 2019-12-20

**Authors:** Kimberly J. Ornell, Jeannine M. Coburn

**Affiliations:** Department of Biomedical Engineering, Worcester Polytechnic Institute, 100 Institute Road, Worcester, MA 01605 USA

**Keywords:** Neuroblastoma, Model, Therapeutic, In vitro, In vivo

## Abstract

Despite advances in cancer therapeutics, particularly in the area of immuno-oncology, successful treatment of neuroblastoma (NB) remains a challenge. NB is the most common cancer in infants under 1 year of age, and accounts for approximately 10% of all pediatric cancers. Currently, children with high-risk NB exhibit a survival rate of 40–50%. The heterogeneous nature of NB makes development of effective therapeutic strategies challenging. Many preclinical models attempt to mimic the tumor phenotype and tumor microenvironment. In vivo mouse models, in the form of genetic, syngeneic, and xenograft mice, are advantageous as they replicated the complex tumor-stroma interactions and represent the gold standard for preclinical therapeutic testing. Traditional in vitro models, while high throughput, exhibit many limitations. The emergence of new tissue engineered models has the potential to bridge the gap between in vitro and in vivo models for therapeutic testing. Therapeutics continue to evolve from traditional cytotoxic chemotherapies to biologically targeted therapies. These therapeutics act on both the tumor cells and other cells within the tumor microenvironment, making development of preclinical models that accurately reflect tumor heterogeneity more important than ever. In this review, we will discuss current in vitro and in vivo preclinical testing models, and their potential applications to therapeutic development.

## Background

Neuroblastoma (NB) is the most common solid, extracranial childhood tumor, accounting for approximately 15% of all childhood cancer deaths [1–3]. Nearly half of all patients are classified as having high-risk disease, portending poor long-term survival despite multimodal treatment [4]. NB is a disease of the sympaticoadrenal lineage of the neural crest, with tumors forming anywhere in the sympathetic nervous system. The tumors most commonly arise in the abdomen (65%), however, they also occur in the neck, chest, and pelvis. Approximately 50% of patients present with evidence of metastasis [5, 6]. Frequent metastasis sites include cortical bone, bone marrow, liver, and lymph nodes [5, 6]. The disease exhibits a broad range of clinical behaviors, making treatment difficult, particularly for high-risk patients [1, 4]. NB typically occurs in children who do not have a family history of the disease, although there are some genetic changes frequently associated with the disease [7]. The most common genetic change is MYCN amplification, which occurs in approximately 20% of patients, and is strongly correlated with advanced stage NB [8, 9]. Additionally, deletions of the short arm of chromosome 1 (1p) are found in 25–35% of patients and can be correlated with MYCN amplification [10–12]. Outside of MYC linked changes, allelic loss of 11q is present in 35–45% of patients and is also associated with high-risk disease features [13, 14].

Treatment strategies for NB are guided by the staging and risk level of the disease. In low risk patients, surgery is frequently curative. Should recurrence of NB occur, it is usually local to the original tumor site and can be managed surgically [14, 15]. Use of cytotoxic therapies is typically avoided due to the high amount of long-term complications [16]. Treatment for patients with intermediate or high-risk NB consists of a multimodal approach including surgical resection, chemotherapy, and radiation [4]. Typical treatment of NB begins with resection of the primary tumor coupled with chemotherapy and radiation to manage the tumor size and facilitate resection [17, 18]. If the tumor is too large for surgical resection, an initial induction treatment with chemotherapy is utilized to shrink the tumor. Chemotherapies typically used include doxorubicin, vincristine, cisplatin, etoposide, and cyclophosphamide [19]. Stem cell rescue can also be used during the consolidation part of the therapeutic regime after high-dose chemotherapy kills the cells within the bone marrow [20].

There are multiple therapeutics currently undergoing preclinical development or in clinical trials for NB. These therapeutics include cytotoxic agents such as topoisomerase 1 inhibitors, radionuclides, retinoids, angiogenesis inhibitors, and tyrosine kinase inhibitors [21–26]. Recently, immunotherapy has emerged as a promising therapy to improve outcomes in patients with advanced stage NB, specifically targeting the highly expressed disialoganglioside GD2. Currently, monoclonal antibodies for GD2 have been clinically approved for therapy in combination with GM-CSF, IL-2, and 13-cis-retinoic acid, while other forms of immunotherapy (e.g. T-cell) are still under development [27–29]. Despite the emergence of novel therapeutics including immunotherapeutics, the prognosis for high-risk patients remains poor.

NB is a heterogeneous cancer, with few distinct subtypes and many different clinical presentations [30]. Development of effective therapeutics is dependent on understanding tumor heterogeneity and the ability to accurately test therapies in a preclinical setting. Preclinical models typically use developed environments (murine or in vitro) to assess how a tumor will respond to therapeutics. The high degree of heterogeneity, lack of consistent genetic markers, and range of prognosis (dependent on stage) makes generating accurate preclinical models difficult. This review highlights current strategies and challenges of in vitro and in vivo NB modeling for preclinical therapeutic testing.

## Main text

### Preclinical murine models

Murine models are frequently used for preclinical testing of therapeutics due to their genetic homology to humans (~ 80%), ability to be genetically manipulated to mimic human diseases, and complex multicomponent environment (e.g stroma, immune cells) [31]. They are advantageous as they provide information regarding therapeutic efficacy that cannot be demonstrated in traditional, less complex in vitro cultures. Murine models are typically a necessary stage before progressing therapies to clinical trials. There are many different types of murine models, including genetically engineered models, spontaneously formed tumors, mice with implanted mouse or human tumors, and, more recently, mixed cell type xenograft models. Each of these models is uniquely suited for preclinical testing of therapeutics. However, there is the still need for further improvement and refinement to drive the development of successful therapeutics.

#### Transgenic mouse models

Transgenic mice, also referred to as genetically engineered mouse models (GEMMs), can be engineered through promotion or addition of genes (knock-in) or inhibition of gene expression (knock-out). Methods of GEMM development have been reviewed elsewhere [32, 33]. Table 1 contains a list of currently used GEMMs for NB. The most widely used GEMM researched for NB is the TH-MYCN model developed by Weiss et al. [34]. These mice overexpress MYCN through a tyrosine hydroxylase promoter. This model was the first to demonstrate that MYCN amplification can drive NB development, identifying the MYCN pathway as a potential therapeutic target. Tumors generated from MYCN overexpressing mice have MYCN protein levels similar to that of the established NB KELLY cell line, known to contain amplified MYCN [34]. Additionally, similar histopathology is observed between MYCN-amplified patient tumor samples and TH-MYCN tumors [35]. This model has been used extensively in preclinical testing for small molecule inhibitors and testing of chemotherapeutics [35–41]. MYCN pathway inhibitors that showed success in vitro in MYCN-amplified cell lines, such as bromodomain and extra-terminal domain protein inhibitors and cyclin-dependent kinase inhibitors, demonstrated similar results in the TH-MYCN model [37, 42, 43]. High MYCN expression has been linked to high levels of angiogenesis. TH-MYCN tumors treated with angiogenic inhibitors, such as the angiogenesis inhibitor TNP-470, demonstrated a high level of response. In treated tumors, intact blood vessels were replaced with hemorrhagic areas containing necrosis and apoptosis [35]. This model has also been used, although to a limited extent, for testing immune checkpoint inhibitors [44]. While the TH-MYCN model has been considered the standard for preclinical modeling of MYCN-amplified tumors, there are still limitations. There is a high rate of tumor incidence in the 129/SvJ background (100% for homozygous mice and 33% for heterozygous mice). However, there is a considerably low rate of tumor incidence in alternative background strains such as BL6 (5% incidence), making crossing with established BL6 GEMMs a challenge [34]. Additionally, distant metastasis frequently occur in the clinical presentation of MYCN-amplified tumors, but are rarely observed in the TH-MYCN model [52]. This model is also limited by the long development time (an average of 65 days), making it difficult for rapid, high-throughput testing [52, 53].

**Table 1:** Table of preclinical genetically engineering murine models

Mouse Model	Advantages	Limitations	References
TH-MYCN	Representative of high-risk NB, high rate of tumor incidence	Long time for tumor development, few metastasis, limited background strain	[34–43]
LSL-MYCN;Dbh-iCre	Better defined transgene insertion then TH-MYCN, high rate of incidence in multiple background strains	Few metastasis, Limited work with preclinical therapeutic testing	[44]
TH-MYCN/CASP8(KO)	Metastasis, high rate of tumor incidence	Altered ECM structure of primary tumor	[45]
TH-MYCN/Trp53(KI)	Inducible p53 loss	p53 mutation more frequently present in recurrences, survival in mice greatly reduced	[46]
ALK (F1174)	Consistent with NB phenotype	Only present in 10% of NB	[47, 48]
TH-MYCN/ALK(F1174)	High tumor incidence, faster tumor growth	Relevance is limited to < 10% patients	[48]
SV40 Tag	Consistent with NB phenotype, high tumor incidence rate, metastasis	All mice die by 28 weeks of age	[49–51]

To address some of the limitations of the TH-MYCN model, a mouse with Cre inducible MYCN expression (LSL-MYCN;Dbh-iCre) was created [53]. This model has a better-defined transgene insertion site allowing tumors to develop at multiple locations in the neural crest, such as the adrenals, the celiac ganglia, and the superior cervical ganglia. Additionally, this model allows for tumor development in multiple mouse strain backgrounds, which is important when combining the TH-MYCN mouse with other cancer relevant alleles [53]. LSL-MYCN;Dbh-iCre mice recapitulates NB histology and molecular expression patterns [53]. These mice are advantageous compared to TH-MYCN mice as the transgene insertion is localized to the commonly used ROSA26 locus, which, when discontinued, causes no phenotypic change in mice. Alternatively, the TH-MYCN mouse primarily inserts into the distal region of chromosome 18, the effects of which have not fully been characterized. While the insertion site has been changed, MYCN expression increased to comparable levels as the TH-MYCN model. Cell lines derived from the LSL-MYCN;Dbh-iCre mouse also respond to the MYCN targeting drugs MLN8327 and JQ1 [53]. This model presents a more defined MYCN-amplified tumor model for preclinical testing, and could prove useful for future testing of therapies aimed at treating high risk MYCN-amplified NB.

In addition to the LSL-MYCN;Dbh-iCre mouse, the TH-MYCN mouse has been genetically modified to incorporate other oncogenes. A cross of a TH-Cre caspase-8 knockout mouse with a TH-MYCN mouse exhibited increased bone marrow metastasis as compared to the TH-MYCN mouse (37% versus 5% incidence) [45]. Loss of caspase-8 does not change the incidence of primary tumors, however, it does change the extracellular matrix (ECM) structure of the primary tumor into a more migratory phenotype with increased collagen 4A2 and laminin α4 as well as increased EMT genes (Snai2, Twist1 and TfpI2). The metastatic propensity of this model could be useful for identifying treatments for metastatic NB. In addition, similar to clinical treatment strategies, the primary tumors in the mice could be debulked and allowed to metastasize. This could allow this model to be used to gain knowledge about the metastatic population and identify therapeutic approaches specifically geared towards those tumors [45].

To mimic the impaired p53 function frequently present in high-risk NB recurrence, a TH-MYCN/Trp53(KI/KI) mouse with a tamoxifen inducible p53ER fusion protein was created [46]. Survival in TH-MYCN/Trp53(KI/KI) mice reduced, and the tumors exhibited decreased radiosensitivity [46]. In addition, when functional p53 was restored to these mice, only 50% regained sensitivity to radiation, suggesting other resistance mechanisms. Additionally, the authors determined that the upregulation of the glutathione S-transferase pathway observed in this model was correlated with poor survival in NB patients [46]. TH-MYCN/Trp53(KI/KI) allografted tumors treated with the glutathione S-transferase pathway inhibitor buthionine sulfoximine regained sensitivity to radiation, suggesting a potential therapeutic strategy for patients with MYCN amplification and impaired p53 function [46].

Other mutations have been examined as a method of inducing NB, such as activation of anaplastic lymphoma kinase mutations (ALK), present in approximately 10% of NB [54]. One model used targeted expression of the most common and aggressive ALK mutation ALK^F1174^. This model exhibited a similar phenotype to NB and syntenic changes similar to those present in clinical NB including 17q gain and MYCN amplification [47]. Preclinically, this model has been used to evaluate drug response to ALK inhibitors and may provide useful insight into treatments for ALK mutated NB. To provide a model comparable to patient tumors that exhibit both MYCN amplification and ALK mutations, the ALK^F1174^ mutation model was crossed with TH-MYCN mice [48]. Mice hemizygotic for both ALK^F1174L^ and MYCN amplification exhibited high tumor penetrance with rapid lethality superior to that observed in MYCN hemizygotes allowing for elucidating the interplay between the ALK and MYCN pathways [55].

Finally, a transgenic mouse line carrying tetracycline inducible simian virus 40 T-antigen (SV40 Tag) has been created using tetracycline responsive elements with a cytomegalovirus promoter and SV40 Tag [49]. These mice die by 28 weeks of age and exhibit bilateral adrenal tumors. When compared to both human adrenal NB and pheochromocytoma, higher similarity to human NB tumors was observed compared to the pheochromocytoma. NB-associated genetic changes were present with upregulation of MYCN, paired-like homeobox 2b, gamma-aminobutyric acid A receptor beta3 subunit, islet 1, and kinesin family member 1A [49, 50]. In addition, when this model was linked to the olfactory marker protein promoter region, it generated a line of mice with highly metastatic tumors originating in the adrenals or sympathetic ganglia. These metastatic tumors were morphologically very similar to clinical NB histologically [51]. While limited preclinical therapeutic testing has been performed with these models, it has the potential to be a promising model for therapeutic testing due to its genetic similarity to NB and ability to mimic metastasis.

GEMMs are advantageous as they utilize mouse homologs of tumorigenic mutations present in patient tumors to mirror clinical tumors. The mouse retains an intact immune system and stroma, allowing for evaluation of therapeutics that target both the tumor and the surrounding microenvironment. They have specific pathway activations, allowing for analysis and targeted therapeutic testing [42, 48, 56]. The genetic changes are constitutively active in mice throughout development or can be induced at a specific developmental stage. This is important as neuroblastoma arises from developing cells in pediatric patients. In addition, transgenic mice with different allele modifications can be crossed in order to study crosstalk between oncogenic pathways [48]. While GEMMs are useful in understanding tumorigenesis and developing therapeutics, there are some drawbacks to these models. They are time consuming and difficult to generate, and while murine pathways share some homology to human pathways, are not a perfect match to humans [31]. Further, a large number of therapies still exhibit a differential response between murine and clinical models [57]. This may be partially due to a lack of control over the modification (e.g. achieving full knock-out) of targeted oncogenes. While more advanced methods such as the CRISPR system have been employed for other cancers and for NB cell lines in vitro, they have yet to be reported on for NB mouse models. In addition, a fundamental problem with many GEMMs is that mutations are frequently not localized to pathological cells and can impact other cells within the mouse [34].

#### Syngeneic mouse models

Syngeneic mouse models, also known as allograft tumor models, utilize tumor cells derived from a mouse of the same genetic mouse strain. Tumor cells can be removed from GEMMs, used to develop cell lines in vitro, then reintroduced into mice of the same strain [58–61]. Tumorigenic cells capable of cell line derivation have been identified in TH-MYCN mice as early as day E13.5 [61]. TH-MYCN tumors from both homozygous and hemizygous mice have been used to develop syngeneic tumors. Whether the mouse was homozygous or hemizygous impacted the cell phenotype and allowed for the creation of different tumor lines. Hemizygous tumors gave rise to cell lines which were phenotypically similar to an N-type NB, expressing high levels of MYCN. Homozygous tumors gave rise to cell lines phenotypically comparable those of hemizygous tumors (N-type high MYCN) and to S-type, adherent NB cells, that exhibited reduced MYCN expression [60]. Interestingly, the cell lines derived from hemizygous tumors also displayed reduced tumorigenicity in a syngeneic model, compared to that of the original tumor phenotype. Further, these cell lines contained many genetic changes present in clinical NB, allowing for syngeneic mice to better represent clinical NB. For example, mouse chromosome homologous to human chromosomes 7 and 12 were gained in one cell line, which has been observed in a subset of clinical cases. Gains homologous to chromosomes 1q and 18q were frequently observed in TH-MYCN derived cell lines, further suggesting that molecular and biological features of NB are present in derived murine cell lines [60]. These murine tumor cells can be transplanted into mice of the background strain, leaving an intact matched immune system and stroma [58–60].

TH-MYCN-derived lines have been transplanted into mice both subcutaneously and orthotopically [58–60]. Injection of tumor lines derived from TH-MYCN tumors in a C57Bl/6 background into C57Bl/6 mice has been used for preclinical testing of immunotherapies [58]. Kroesen et al. demonstrated the relevance of this model for immunotherapy testing due to the tumors expressing similar surface marker profiles (low HLA molecules and the presence of NKG2D activating ligand Rae1), and containing similar resident immune populations [58]. In addition to TH-MYCN-derived tumors, other tumors derived from murine cell lines, such as neuro-2a (spontaneous NB from strain A albino mice, C1300 derived), TBJ (C1300, a strain A/J spontaneous tumor), 9464D (TH-MYCN on a C57Bl/6) and NXS2 (created from C1300 tumors) have been engrafted both subcutaneously and orthotopically for testing of immunotherapeutics [62–64]. In particular, preclinical testing of GD2-targeting immunotherapeutics, both individually and in combination with IL-2, and examining the impact on other immune components has been performed using these models [58, 59, 64–66].

Syngeneic transplantations of murine cells are advantageous as tumorous cells can be engrafted in mice with a non-genetically modified matching immune system and stroma [58, 59]. Unlike transgenic mouse models, the genetic mutations are confined to the transplanted tumor cells. In addition, there are typically an abundance of cells for transplantation, which allows for large scale therapeutic testing [58]. Further genetic modification of cells in vitro can also be performed to either add tumor relevant pathway modifications or to add markers to improve cell visualization such as fluorescent or luciferase labels [63, 67, 68]. Potential modifications to cells will be discussed further in the in vitro model’s section. Disadvantages of syngeneic models include inconsistency in tumor engraftment and the use of the murine system. Similar to GEMMs, engrafted tumors are frequently based on a single oncogenic based mutation as compared to the heterogeneity exhibited in clinical tumors [52].

#### Xenograft models

Human cells have been engrafted into mice for preclinical testing and understanding mechanisms of NB development. Xenograft models can be generated via subcutaneous or orthotopic injection of human NB cells. Tumors developed in these studies are advantageous compared to those developed in GEMMs or in syngeneic models as they more closely mimic a primary human tumor and are better predictors of clinical outcomes [52]. A table of different human cell lines commonly xenografted into mice can be found in Table 2. Use of cell lines with different genetic profiles allows for the formation of tumors with different phenotypes and growth rates [52, 69]. Additionally, these tumors mimic some of the heterogeneity observed in patient tumors [52]. They are typically easier to generate than tumors generated using primary patient-derived cells, and allow for large scale studies, as cell lines can be scaled up in vitro before engraftment. However, they require the use of immunocompromised mice (typically lacking T-cells) for engraftment and survival, which provides a less realistic tumor microenvironment and limits the conclusions that can be made when testing immunotherapies. Both orthotopic and subcutaneous tumors have been used extensively for preclinical testing of therapeutics. Therapeutic testing has extended to chemotherapy, radiotherapy, small interfering RNA, antisense oligonucleotides and pathway inhibitors as well as drug delivery methods such as nanoparticles and drug-loaded scaffolds or films [56, 69, 102–108]. In addition to subcutaneous and orthotopic injection, Seong et al. used intracardiac injection xenograft models to identify NB sub-populations with a higher metastatic potential. Metastatic populations and phenotype differences correlated with genetic changes representing utility for these metastatic cells in identifying new therapeutic targets [109].

**Table 2:** Frequently used human NB cells lines for preclinical testing

Cell Line	MYCN Status	ALK Mutation	P53 mutation	Preclinical Testing References
KELLY	Amplified	WT	WT	[69–75]
CHP-212	Amplified	WT	WT	[76, 77]
SKNAS	Non-Amplified	WT	H168R	[76, 78–86]
SH-SY-5Y	Non-Amplified	F1174 L	WT	[70, 71, 75, 78, 87–89]
IMR-32	Amplified	WT	WT	[80, 88, 90, 91]
IMR-05	Amplified	WT	WT	[37, 86, 89, 92]
LA-N-5	Amplified	R1275Q	WT	[71, 93, 94]
NB-1	Amplified	WT; Amplified	WT	[95, 96]
SK-N-BE(2)	Amplified	WT	C135F	[75, 78, 83, 89, 86, 97]
SK-N-BE(2)-C	Amplified	WT	C135F	[78]
CHP-134	Amplified	WT	WT	[78, 89, 98, 99]
SK-N-DZ	Amplified	WT	R110L	[82, 84, 85]
NB-1691	Amplified	WT	WT	[83, 100, 101]

Borriello et al. evaluated the impact of the heterogeneous microenvironment. NB cell lines with and without bone marrow-derived mesenchymal stromal cells (BM-MSCs) and cancer-associated fibroblasts (CAFs) taken from NB patients were injected subcutaneously and just below the renal capsule [110]. Engrafted tumors were treated with the chemotherapeutics etoposide, etoposide with ruxolitinib (JAK2/STAT3 inhibitor), and trametinib MEK/ERK1/2 (inhibitor). These inhibitors were chosen as CAFs and BM-MSCs have activated STAT3 and ERK1/2 pathways, which contributes to drug resistance. No difference in response to etoposide between NB cells and NB cells with BM-MSCs and CAFs was observed. However, response to etoposide by NB cells and NB cells with BM-MSCs and CAFs was enhanced when combined with ruxolitinib and trametinib. In the NB cell alone tumors, murine CAFs were identified within the tumor, potentially explaining the similarity in response.

While cell lines injected into mice are typically passaged in vitro, many studies have shown that traditional in vitro culture methods significantly impact the cell genotype and phenotype. This may be due to the cells adapting to the tissue culture environment and the lack of in vivo relevant signaling. Instead, cells in tissue culture rely on culture medium, with potential adverse effects, specifically related to fetal bovine serum. Fetal bovine serum is frequently used as a source of hormone factors, essential nutrients, and growth factors needed for a stable growth environment [111]. However, growth with serum has been demonstrated to lead to cellular differentiation and genetic changes, causing the cells to stop mimicking their original clinical phenotype and increase their drug sensitivity [112, 113].

Since NB is an orphan disease, a limited number of patient-derived tumor specimens are available. Patient-derived xenografts (PDX) are typically taken directly from patients and passaged by subcutaneous or orthotopic implantation of primary tumor pieces or injecting tumor cells into mice. A list of current PDX tumor cells and suppliers can be found in Table 3. Passaging the tumor as a xenograft eliminates in vitro adaptations often observed with sub-cultured cell lines [114, 115]. Braekeveldt et al. and Stewart et al. demonstrated that PDX tumor cells orthotopically grown shared molecular characteristics with primary NB cells, retained classic NB markers, and spontaneously metastasized in murine models [114, 115]. Increased infiltration and distant metastasis were observed with orthotopically injected PDX cells as compared to orthotopically injected cell lines. In addition, hallmarks of the microenvironment such as vascular infiltration, CAFs, and tumor-associated macrophages (TAMs) with an M2 phenotype were observed in orthotopic PDX tumors [116]. Continuous xenograft transplantation has also been used to identify genetic changes that tumors undergo during metastasis [117]. Regarding patient-derived tumor cells propagated in vitro*,* generating non-adherent cell lines by culturing with basic fibroblast growth factor, epidermal growth factor, and B27 without serum more closely mimics primary cell lines both in vitro and in vivo [118].

**Table 3:** Available NB PDX Cell Lines and Sources

PDX Line	Stage of Tumor	Age of Patient (Yr.M)	MCYN Status	p53 Status	Organization
NB-SD	4	1	Amp	Mut	Pediatric Preclinical Testing Program
NB-1771	4	2.1	Amp	Mut	
NB-1691	4	1.9	Amp	WT	
NB-EBc1	4	2.6	Non Amp	WT	
CHLA-79	4	2	Non Amp	WT	
NB-1643	4	1.7	Amp	WT	
NB-1382	3	3.5	Amp	N/A	
IGR-NB8	3	5.0	Amp	N/A	Insitut Curie
IGR-N835	4	2.0	Amp	N/A	
MAP-IC-A23-NB-1	L2	2.6	Non Amp	N/A	
MAP-GR-B25-NB-1	4	4.0	Amp	N/A	
MAP-GR-A99-NB-1	4	1.10	Amp	N/A	
HSJD-NB-011	4	2.6	Amp	N/A	
SJNBL012407_X1	4	0.1	Amp	N/A	Children’s Solid Tumor Network
SJNBL013761_X1	4	3.0	Non Amp	N/A	
SJNBL013762_X1	4	1.3	Amp	N/A	
SJNBL013763_X1	2B	2.0	Amp	N/A	
SJNBL015724_X1	4	2.0	Non Amp	N/A	
SJNBL046_X	4	2.0	Amp	N/A	
SJNBL108_X	4	3.0	Non Amp	N/A	
SJNBL046145_X1	4	2.0	Non Amp	N/A	
SJNBL046148_X1	4	1.11	Amp	N/A	
SJNBL047443_X1	4	12.0	Non Amp	N/A	
PDX-1	4	1.4	Amp	N/A	Lund University
PDX-2	4	2.2	Amp	N/A	
PDX-3	3	2.9	Amp	N/A	
PDX-4	4	4.9	Amp	N/A	
PDX-5	4	2.4	Non Amp	N/A	
PDX-6	2B	12.0	Amp	N/A	
COG-N-415x	Unknown	Unknown	Amp	WT	Children’s Oncology Group
COG-N-440x	Unknown	Unknown	Amp	WT	
COG-N-453x	Unknown	Unknown	Amp	WT	
COG-N-471x	Unknown	Unknown	Amp	WT	
COG-N-496x	Unknown	Unknown	Amp	N/A	
COG-N-519x	Unknown	Unknown	Amp	N/A	
COG-N-534 m	Unknown	Unknown	Non Amp	N/A	
COG-N-549x	Unknown	Unknown	Non Amp	N/A	
COG-N-557x	Unknown	Unknown	Amp	N/A	
COG-N-573x	Unknown	Unknown	Amp	N/A	
Felix (COG-N-426)	Unknown	Unknown	Non Amp	N/A	
CHLA-90	4	8	Non Amp	N/A	
CHLA-136	4	3	Amp	N/A	

*Amp* Amplified, *Mut* Mutation, *WT* Wild-type, *N/A* Not Available

PDX models have been used to evaluate standard of care chemotherapeutics and targeted therapeutics [115]. While PDX tumors are the gold standard for xenograft models, there are still many limitations. The time to establish tumors is long and generating enough consistently sized tumors for large scale therapeutic studies is difficult. In addition, PDX cells are injected into immunocompromised mice, limiting their effectiveness for testing of immunotherapies [119]. In vivo, PDX cells rely on the mouse microenvironment, which does not completely mimic that of a human and confounds potential stromal interactions [116].

#### Xenografted tumors in “humanized” mice

A major limitation of xenograft models is the use of immunocompromised mice that lack a fully functional immune system. As more immunotherapies are being developed, identification of preclinical models for testing them is critical. Recently, immunodeficient mice with humanized immune systems have emerged as a method to examine xenografted tumor growth with an engrafted human immune system. These humanized mice (HM) are developed to investigate the interactions between tumor cells and immune cells. There are several methods of developing HM, the most basic of which consists of direct injection of human peripheral blood into immunocompromised mice [116]. Alternatively, stromal tissue can be injected alongside tumor tissue, resulting in an active immune population [120]. More commonly, human hematopoietic stem cells and/or precursor cells (CD34+ or CD133+) are injected into the bone marrow of irradiated immunocompromised mice, allowing for the generation of immune cells including T cells, B cells, and macrophages [121]. This method is advantageous as a patient’s own marrow or blood could be injected into the mouse, allowing for matching between the immune system and tumor. However, successful use of this method has not been reported yet for NB. While the method of hematopoietic stem cell injection is extremely promising, there are still many components that need to be developed. These models still retain mouse stroma and cytokines, which has the potential to prevent complete immune cell differentiation including T cells and B cells [121]. Furthermore, these models have been shown to exhibit antigen-specific immune responses [122, 123]. The development of accurate humanized mice represents the future for effective pre-clinical therapeutic development.

### Preclinical in vitro models

While murine-based systems are the primary method for preclinical testing, advances in tissue culture techniques and in vitro systems are promising for creating accurate NB models. Furthermore, the high cost of murine models as well as cross species pathways and microenvironment differences makes accurate, high-throughput screening challenging. In vitro models encompass a wide range of systems, including traditional adherent monolayer cells, cells grown in 3D suspension cultures (spheroids), and more complex tissue engineering approaches. In addition, they allow for testing of cell response or cell-cell communication in a more controlled manner (e.g. control of cell confluence, ratio of different cell types). While in vitro systems are already used for screening of therapeutics prior to in vivo studies, advances in tissue engineering approaches are creating more accurate models that may better predict clinical efficacy.

#### Monolayer in vitro systems

Traditional in vitro models consist of commercially available or lab-derived cell lines adherent to polystyrene dishes, typically grown in the presence of fetal bovine serum, nutrients, and antibiotics. Monolayer culturing is the most common method of evaluating therapeutic efficacy, primarily due to the higher number of cells that can be generated, which allows for rapid screening of many compounds. In addition, these cells can be modified at the genetic level to evaluate the impact of pathway changes on therapeutic efficacy. Methods of inducing gene changes, including transfection, transduction, and more recently using CRISPR systems, have been previously reviewed [124–126]. Genes that have been identified as potential mediators in NB pathways can then be evaluated through knockdown, overexpression, or direct targeting using pathway inhibitors.

MYCN-amplified cell lines have been useful in identifying many proteins and genes that either contribute to or are associated with MYCN. Park et al. determined that high expression of protein arginine methyltransferase 5 (PRMT5) was strongly associated with MYCN amplification. Use of short-interfering RNA to reduce expression of PRMT5 decreased MYCN expression and caused cell death in MYCN-amplified cell lines [127]. Ambrosio et al. identified lysine-specific demethylase 1 (LSD1), a histone modifier, as a transcriptional modulator of NDRG1 (N-Myc Downstream-Regulated Gene 1, a metastasis suppressor). In both in vitro models and patient samples, high levels of LSD1 correlated with low levels of NDRG1 [128].

RNAi and CRISPR screens have been useful in identifying genes that could be targets for therapeutic regimes. In a kinome-wide RNAi screen, Shen et al. targeted protein kinases and kinase associated genes to identify sensitizing and inhibitory kinases to HDAC8 inhibitors. Knockdown of ALK sensitized NB cells to HDAC8 inhibitors. This was further confirmed through combinatorial treatment of NB tumors with crizotinib, an ALK inhibitor, and HDAC8 inhibitors resulting in increased cell death [129]. CRISPR-Cas9 screening of MYCN-amplified NB cells by Chen et al. demonstrated cellular dependency on the PRCR2 complex, specifically EZH2. MYCN binds at the EZH2 promoter, repressing neuronal differential of NB cells, which promotes a more tumorigenic phenotype. This was further confirmed through genetic and pharmacological suppression of EZH2, which inhibited NB growth. These screens are useful in identifying key pathways that could be therapeutically targeted in NB [130].

In addition to screening through genetic modifications, screens of high numbers of cell lines and therapies can be conducted in vitro. Mahoney et al. screened 482 cell lines with metabolic inhibitors [131]. Neuroendocrine cells, specifically NB cells, showed a higher sensitivity to NB-598, an inhibitor of enzyme squalene epoxidase (SQLE). This suggests that targeting this pathway may have therapeutic potential. Similarly, Michaelis et al. screened 321 cancer cell lines (from 26 different types of cancer) for response to flubendazole (inhibitor of microtubule function) [132]. NB was identified as highly sensitive to flubendazole, reducing viability of 140 NB cell lines. Large scale screens have the potential to identify novel therapeutics for NB.

#### Monolayer co-culture models

The NB tumor microenvironment is composed of multiple cell types including vascular cells, tumor-associated macrophages, fibroblasts, T-cells, natural killer (NK) cells, and others [133]. Each cell type has the potential to influence NB phenotype based on cell-cell interactions, paracrine signaling and secreted factors. Hashimoto et al. co-cultured NB cells with two prominent microenvironment cells, fibroblasts and macrophages. Consistent with clinical results that correlated areas of fibroblasts with aggressive NB phenotype, co-culturing with fibroblasts increased NB cell proliferation [133]. In addition, peripheral blood macrophages were co-cultured with NB cells directly and indirectly using the NB cell-conditioned medium NB cell-conditioned medium transitioned the macrophages into a pro-tumor survival M2 phenotype, or TAM phenotype, suggesting a crosstalk between NB cells and macrophages supporting tumor progression. Indirect co-culture of NB cells in macrophage medium increased tumor invasiveness (through a Matrigel based invasion assay) likely through CXCL2 secretion [133]. Direct co-culture of NB cells and macrophages did not result in an increase in NB proliferation; however, it did enhance the invasiveness of NB cells. In co-culture with NB cells, both macrophage and fibroblasts exhibited enhanced invasion.

Borriello et al. co-cultured NB cells with fibroblasts derived from MSC cells. They observed that fibroblasts induced a pro-tumorigenic effect on NB cells, including increased proliferation and inhibited apoptosis [110]. Chemoresistance to etoposide and melphalan was evaluated using chemosensitive and chemoresistant NB cell lines co-cultured with fibroblasts. It was determined that co-culturing significantly reduced responsiveness of both the NB cell lines and the fibroblasts to chemotherapy. This suggests that presence of fibroblasts in the tumor bed may contribute to chemoresistance. The authors also determined that these effects do not require cell-to-cell contact but are likely due to soluble factors, many of which have convergent activity in the STAT3 and ERK 1/2 signaling pathways [110].

To evaluate the effect of ECM components on self-organization in co-cultures, Rizvanov et al. co-cultured NB cells MSCs on different surfaces including poly-l-lysine, fibronectin, gelatin, collagen I, and Matrigel to examine the surface effect on cell phenotype [134]. No phenotypic differences were observed between non-coated surfaces and surfaces coated with poly-l-lysine, fibronectin, gelatin, or collagen I. In these culture conditions, cells organized into distinct patterns with channels of MSCs and islands of NB cells, comparable to a tumor. However, when cultured on Matrigel, MSCs organized themselves into a dense core with a surrounding ring of NB cells. The authors suggested that this phenotype was more representative of metastatic tumors and could be used as a potential model for metastasis. In addition, exposure of this co-culture model to oxidative stress through the addition of hydrogen peroxide demonstrated that the presence of MSCs increased NB cell viability [134]. As oxidative stress is one of the primary death mechanisms of radiation therapy, this finding implies that this culture system is more mimetic of an in vivo resistant tumor [134].

Co-culturing of NB and NK cells is frequently used as part of an antibody-dependent cell-mediated cytotoxicity assay for testing of immunotherapies. The NK cells induce lysis of NB cells in the presence of antibodies [135]. Similar studies have been carried out to test therapeutic efficacy with leukocytes, peripheral blood mononuclear cells, and granulocytes [136, 137]. These systems have been used to test out combination therapies of cytokines or retinoids with immunotherapies [138, 139]. However, there has been little work done with longer-term culture of these immune cell populations and characterization of the impact of co-culture on tumor cells and immune cells. As immune cells are present in NB tumors, further development of these co-culture models may be critical to developing better therapeutic strategies.

NB cells have also been cultured with hepatocytes, as NB frequently metastasizes to the liver [140, 141]. The authors observed resistance to apoptosis by the NB cells, induction of apoptosis in the hepatocytes, and an increase in VEGF secretion. The hepatocytes induced overexpress of Bcl-2 by the NB cells, thereby reducing NB apoptosis and establishing Bcl-2 as a therapeutic target for NB liver metastasis. Interestingly, studies focusing on VEGF secretion demonstrated that expression of VEGF receptors is highly heterogeneous across NB lines, which likely extends to patient tumors [140]. It is therefore critical to examine the expression of each individual tumor when investigating the use of anti-VEGF therapies [140, 141].

Co-culture systems also enable the investigation of cell migration in the presence of other cell types, as commonly evaluated using transwell plates. NB cells in transwell systems have been used to examine the impact of NB cell MYCN-amplification level on migration of human umbilical vein endothelial cells (HUVECs). HUVEC migration was proportionate to MYCN-amplification level. In addition, it was demonstrated that the efficacy of a PI3K inhibitor NVP-BEZ235 (as angiogenesis is regulated by PI3K) was dependent on MYCN. This suggests a link between angiogenesis, the PI3K pathway, and MYCN in NB [142]. Additional studies have demonstrated that growth of HUVEC in medium conditioned by NB cells induced vessel angiogenesis and upregulation of VEGF and IL-8 [143].

Co-culturing NB cells with other cell types present within the tumor microenvironment such as fibroblasts, immune cells, and cells present at metastatic sites allows for understanding of the impact of tumor microenvironment on NB phenotype [133]. These culture systems allow for an increase in understanding of tumor heterogeneity as well as critical tumor signaling pathways. For preclinical therapeutic testing, co-culture systems provide an opportunity to better understand tumor escape as mediated by signaling factors secreted from neighboring cells and how therapies influence non-tumor cells. Incorporating additional microenvironment stress components, including hypoxia and mechanics, would add additional complexity and relevance for drug development.

#### 3D in vitro models: spheroid

Growth of NB cells in spheroids has been used as a preclinical model*,* as spheroids have been suggested to more accurately mimic the clinical phenotype as a drug screening model [114, 116, 118]. NB spheroid cultures can be generated using low or non-attachment culture dishes, coated plates or dishes, or the removal of serum from the medium [144–147]. Compared to cells grown in monolayer culture, spheroid cultures exhibited increased expression of metabolic markers, cell stress response proteins, cell structure proteins, and transport polypeptides [148]. Sidarovich et al. used spheroids for high throughput drug screening of over 300 FDA-approved anti-cancer compounds or compounds undergoing clinical trials [149]. From this screen, the authors identified, and later evaluate in vivo*,* two compounds, ponatinib and axitinib, as potential new therapies for NB based on toxicity, molecular target, and side effects [149].

Spheroids are advantageous as once they reached a critical size of > 100 μm, they begin to exhibit microenvironment changes due to nutrient and oxygen gradients [146]. Changes in nutrient and oxygen gradients induce therapeutic resistance through upregulation of pro-survival and tumor promoting pathways [146]. Growth of NB cell lines and patient-derived tumor cells as non-adherent spheroids demonstrated retention of cellular phenotype more closely resembling primary tumors [118]. Additional advantages of spheroids include altered diffusion and ECM deposition that has the potential to impact therapeutic efficacy [144].

Culturing as spheroids demonstrated selectively for tumor-initiating cells [150]. Coulon et al. evaluated spheroid formation of serially passaged PDXs and found that only the bone marrow-derived metastatic cells (the patient equivalent of stage 4) were able to generate spheroids. The generation of spheroids suggests that the metastatic cells have a high degree of self-renewal and are likely enriched with a cancer stem cell population [151]. This finding is important as it supports the use of spheroids for testing drugs that are associated with tumor stem cells such as Notch and WNT [152, 153]. MYCN-amplified tumor cells exhibit a higher propensity for spheroid formation than non-MYCN-amplified cells [151]. The ability to form spheroids is directly dependent on the cellular differentiation status, or stemness. Treatment with 13-*cis*-retinoic acid, a differentiation agent that induces a neuronal phenotype, inhibited spheroid formation [154].

Gransbury et al. fabricated spheroids of different diameters (ranging from 50 to 800 μm) to evaluate different microenvironments [146]. Using the spheroid culture, the authors were able to examine different levels of hypoxia, diffusion, and redox state, giving them further insights into the therapeutic potential than otherwise possible with 2D culture models [146]. Two different cancer therapies were identified: NAMI-A and KP1019 which are Ru^III^-based anti-metastatic and cytotoxic drugs that are administered in a non-active form and subsequently become activated under oxidative environments. Cuperus et al. used spheroid cultures to study fenretinide, which has been shown to induce apoptosis through retinoic acid and reactive oxygen species dependent pathways [155]. In combination with buthionine sulfoximine, an inhibitor of glutathione synthesis, fenretinide reduced proliferation and induced apoptosis both in monolayer and in spheroids.

Spheroid culture systems have been used to model drug diffusion challenges and develop drug delivery systems with improved tumor penetration. Sagnella et al. used spheroid culture of NB to evaluate the therapeutic potential of EDV™ nanocells – a bacterially-derived drug delivery system consisting of nonviable cells that are ~ 400 nm in diameter. The authors demonstrated that the EGFR-targeted nanocells enhanced penetrance of doxorubicin compared to non-targeted doxorubicin loaded nanocells and doxorubicin without a delivery vehicle, resulting in increased apoptosis. These findings were confirmed in an orthotopic xenograft model [156].

Spheroids have been directly compared to monolayer cells to determine efficacy of different forms of radiotherapy [157]. Cunningham et al. demonstrated that the Auger electron-emitting conjugates ^123^I-meta-iodobenzylguanidine and ^125^I- meta-iodobenzylguanidine and the alpha-emitting conjugate ^211^At- astatobenzylguanidine were highly toxic to cells grown in monolayer and in small spheroids. In larger spheroids, the Auger electron emitters were relatively ineffective. However, a beta-emitting conjugate ^131^I-astatobenzylguanidine was highly effective in large spheroids. This work highlighted how spheroids may assist in identifying therapies that may be more successful clinically, either for killing macroscopic tumors [157]. Spheroids have also been used to evaluate specific pathway inhibitors such as multikinase inhibitors and oxidative phosphorylation inhibitors [158, 159].

Spheroid cultures are an important part of preclinical testing. They are currently the most widely used approach to bridge the gap between two-dimensional cell culture and the in vivo tumor microenvironment [147]. Growth in spheroids exhibits phenotypes better resembling in vivo tumors [119]. In addition to a higher degree of mimicry to in vivo tumors, spheroids are advantageous as they allow for rapid preclinical testing [147]. They also exhibit cell-cell contact similar to that of an in vivo tumor and exhibit similar diffusion limitations for nutrients and therapeutics [114, 116, 118]. Limitations to spheroid tumors include heterogeneity in sizes, a necrotic core in large spheroids, and lack of additional environmental components such the stroma and immune cells [160, 161]. Non-uniform spheroid generation results in varying diffusion gradients, making properly controlled experiments challenging. Frequently tumors in vivo exhibit a hypoxic core, with the necrotic cells secreting factors which induce angiogenesis, thus preventing the tumors from becoming overly necrotic. Angiogenesis does not occur without a vascular component in the spheroid cultures, so care must be taken to avoid large regions of necrosis. Stroma and immune cells are critical components of the tumor microenvironment and can impact oncogenic pathways and therapeutic efficacy. Co-culture with stroma and immune components in spheroids has been demonstrated to be effective for other cancer types, and presents a unique opportunity to mimic NB the tumor microenvironment [162–165].

#### Hydrogels and scaffolds for 3D tumor growth

Limited work has been done with NB growth in 3D outside of spheroid cultures. However, 3D growth of NB cells can be achieved using a broad range of scaffold and or hydrogel materials. ECM hydrogels such as collagen I and Matrigel have been used to mimic tumor ECM and provide a backbone for 3D tumor growth. These 3D matrices have the potential to impact gene expression and cell morphology [166]. Li et al. used microarray analysis to demonstrate differences in gene expression in cells grown in monolayer, collagen I hydrogels, and Matrigel hydrogels [166]. All 3D culture conditions induced morphological differences as compared to monolayer. Cells grown in collagen I exhibited longer neurites than those grown in Matrigel, likely due to the fibrils present in the collagen. This study focused on genes associated with morphology and neurite outgrowth; studies evaluating the impact on key NB tumor pathways have not been performed.

Mitchell et al., used spheroids embedded in collagen hydrogels to evaluate the invasive behavior of NB [167]. Mixed cell population of NB cells, neuronal type NB cells and stromal type NB cells, exhibited a heterogeneous invasive population. Crosstalk between both cell types was identified where neuronal type NB cells decreased invasion of stromal type NB cells and stromal type NB cells enhanced invasion in neuronal types NB cells [167]. This could be useful in identifying which cell populations to target therapeutically to decrease metastasis.

Studies have suggested the presence of ECM molecules as well as growth in 3D can impact the responsiveness to therapeutics [168]. Mitchell et al., evaluated siRNA-targeting Rac on both single cell suspensions and cells suspended in 3D collagen hydrogels. Cell lines with different morphologies, stromal, neuronal, or combination, were evaluated. As Rac inhibition is most effective in cells with elongated, invasive morphology, studies in 3D were critical to identifying differences in invasion and morphology for determining therapeutic efficacy [168].

Non-hydrogel-based scaffolds typically consist of porous or fibrous materials (either synthetic or biological-derived) that may mimic structures present in vivo. They are advantageous as they have tunable degradation based on material properties or material choice, can be functionalized to mimic the native environment, and provide control over spatial organization. Scaffolded approaches have been used to model other cancer types, but limited scaffolded approaches have been utilized for NB. Curtin et al. used lyophilized collagen I/glycosaminoglycan and collagen I/nanohydroxyapatite scaffolds for culturing of KELLY NB cells and a cisplatin-resistant KELLY cell derivative [169]. The NB cells exhibited reduced growth in 3D as compared to monolayer culturing, which is consistent with previously demonstrated results in other cancer cell lines [170, 171]. The cisplatin-resistant cell line exhibited increased proliferation in the collagen/hydroxyapatite scaffold as compared to the collagen/glycosaminoglycan scaffold. Response to cisplatin was evaluated in monolayer, 3D culture, and an in an orthotopic xenograft model. Both 3D culture models exhibited similar chemosensitivity to the orthotopic in vivo model, with a reduced response observed as compared to the monolayer culture. Using these scaffolds, KELLY NB cells grown in 2D and 3D were used for evaluation of liposomes delivering miRNA for therapeutic gene silencing [169]. Unlike the chemotherapy studies, miRNA exhibited similar effects in 2D and 3D highlighting the potential usefulness of miRNA as a therapeutic.

Scaffold based studies have also been used for biomechanical modeling. One scaffold-based study used graphene-augmented nanofiber scaffolds to determine the impact of an “out-of-comfort” nanobiomechanical environment for NB cells [172]. Growth on highly aligned graphene fibers changed the morphology from flat to a more rounded shape as the cells enveloped the fibers. In addition, increased gene expression of pro-migratory and pro-invasion markers were observed [172]. This represents a potential system to examine the more migratory or more metastatic NB cells, and develop therapeutics aimed at effectively inhibiting those cells.

3D scaffold-based modeling has been explored for other pediatric blastoma and similar pediatric cancers. For example, polymeric poly (lactide-co-glycolide) nanoparticles have been used to generate 3D cultures of retinoblastoma cells. Etoposide and doxorubicin loaded nanoparticles induced higher cytotoxicity towards 2D cultured cells as compared to 3D cultured cells. The authors correlated this to a decreased drug exposure in 3D cultured cells as compared to 2D, likely due to diffusion barriers [173]. Electrospun poly(ε-caprolactone) microfiber scaffolds have been used to generate models of Ewing sarcoma [174]. Using these models, cells grown in microfiber scaffolds exhibited reduced proliferation as compared to 2D cultured cells. Cell growth on the microfiber scaffold was comparable to xenograft growth, as were levels of relevant pathways such as IGF-1R signaling and mTor [174]. Ewing sarcoma cell lines and PDX cells have been grown on a porous matrix composed of freeze-dried type I collagen and hyaluronic acid meant to mimic both mechanical and biological cues present in the body. Both the Ewing sarcoma cell lines and PDX cells demonstrated increased drug resistance and closer resemblance of in vivo tumors [175]. Scaffold-based approaches could be applied to preclinical NB modeling. These approaches are advantageous as scaffolds can be fabricated from materials that mimic the native tumor ECM (both chemically and mechanically). In addition, use of layered scaffold models with multiple cell types can be used to model different components of the tumor architecture.

### 3D co-culture models

3D models can be expanded to include multiple cell types found in the tumor microenvironment. Villasante et al. used a tissue engineered model consisting of sheets of HUVEC cells and NB cells stacked to reach a total height of ~ 100 μm placed on a “vascular bed” made of collagen, fibrin, and HUVEC cells. The stacked vascular bed was placed on a collagen gel with microchannels to mimic vessel-like structures within NB [176]. This system was cultured in a perfusion bioreactor to mimic the in vivo environment. The therapeutic potential of isotretinoin was evaluated using this model. Isotretinoin blocks cell proliferation and reduces tumor vasculature in vivo. Isotretinoin increased cellular apoptosis, and decreased mRNA levels of NB markers MYCN and GLI1. In addition, isotretinoin weakened and disassembled the vascular networks by blocking cell-to-cell adhesions. Populations of both cancer cells and vascular cells resistant to isotretinoin were identified. Further characterization of resistant cells identified an increase in SOX2 expression in the resistant population. This correlation had not previously been identified using conventional 2D culture [176].

NB cells and MSCs have been co-encapsulated inside collagen I microspheres to investigate the impact of the stromal environment on NB growth [177]. The MSCs were used as they exhibit a fibroblast like morphology, resembling CAFs in vivo. The MSCs induced increased NB proliferation, suggesting that the stromal component has a direct impact on tumor growth. The cultured NB cells and MSCs exhibited a rosette-like phenotype, resembling that of clinical NB. MMP9-expressing cells were found primarily on the periphery of the microspheres, which is the more migratory region. Previous work with these microspheres identified a hypoxic core, which could be evaluated with the NB model to mimic the hypoxic core frequently found in clinical NB tumors [178].

Multicellular models grown in 3D are an emerging trend in tissue engineering in an attempt to understand the complex tumor microenvironment. These models remain largely unexplored for NB; however, preliminary studies suggest that they can provide insightful information about tumor pathway crosstalk and potential therapeutic efficacy. The heterogeneity in NB, both within the tumor microenvironment and across individual tumors, represents a challenge to effective therapeutic development. In vitro 3D culture of NB cells with relevant microenvironment cells would allow for elucidation of critical pathways and mechanisms of resistance that exist in vivo.

## Conclusion

Neuroblastoma is a heterogeneous disease, both in clinical presentation and prognosis. Understanding of critical pathways in disease progression and development of effective preclinical therapies for NB remains a challenge. Murine models, including GEMM, syngeneic, and xenograft have been developed for therapeutic testing, particularly geared towards mimicking high-risk phenotypes. However, challenges remain as therapeutic development trends toward immunotherapies and a mouse capable of combining a human NB tumor with an intact immune system has not been created. The future of this likely lies within humanized immune mouse models. These have the potential to use a mouse as a vehicle to evaluate a human tumor, with an intact human immune system.

Tissue engineering provides a promising approach for development of systems capable of high throughput therapeutic evaluation using multicellular systems. The growth of cells in 3D allows for diffusion gradients of nutrients, oxygen, and therapeutics similar to those found in vivo. For development of effective models, it is critical to incorporate multiple cell types (stromal, vascular, and immune) in an environment capable of mimicking relevant diffusion limitations. Patient derived tumors are the most representative of the heterogeneous tumor phenotype. In the future, combining patient tumors with patient-derived stroma and immune cells may achieve a more accurate model for preclinical therapeutic testing.

## Data Availability

Data sharing not applicable to this article as no datasets were generated or analyzed during the current study.

## Abbreviations

ALK: Anaplastic lymphoma kinase
CAF: Cancer associated fibroblast
ECM: Extracellular matrix
GEMM: Genetically engineered mouse model
HM: Humanized mice
MSCs: Mesenchymal stem cells
NB: Neuroblastoma
NK: Natural killer
PDX: Patient-derived xenografts
SV40 Tag: Simian virus 40 T-antigen
TAM: Tumor associate macrophage
